# Pain originating from the sacroiliac joint is a common non-traumatic musculoskeletal complaint in elite inline-speedskaters - an observational study

**DOI:** 10.1186/2045-709X-20-5

**Published:** 2012-03-09

**Authors:** Alexander Ruhe, Tino Bos, Arne Herbert

**Affiliations:** 1 Deutsche Gesellschaft fuer Sportchiropraktik, Porschestrasse 1, 38440 Wolfsburg, Germany; 2 Praxis fuer Chiropraktik, Wolfsburg, Germany; 3 Praxis fuer Chiropraktik, Aachen, Germany

**Keywords:** Speedskating, Non-traumatic, Sacro-iliac joint, Sport-specific, Pain

## Abstract

**Study design:**

Observational study

**Objectives:**

To investigate common non-traumatic musculoskeletal complaints of the low back in elite inline-speedskaters of the German national team.

**Summary of background data:**

Traumatic injuries associated with falls or collisions are well documented in speedskaters but so far no studies have investigated non-traumatic low back pain. Previously, the sacroiliac joint was suspected as a frequent origin of complaint, we aimed to investigate this assumption.

**Methods:**

Two chiropractors examined elite inline-speedskaters of the German national team during three sports events between summer 2010 and 2011. A test cluster of five provocative tests for the sacroiliac joint was selected based on reliability and validity.

**Results:**

A total of 37 examinations were conducted on 34 athletes with low back pain during the three sport events. The reported pain intensities ranged from mild to moderate pain (VAS 23.4 ± 13.4 to 35.1 ± 19.2). About 90% of cases showed involvement of the SI joint of which again 90% presented with left sided symptoms.

**Conclusions:**

Non-traumatic complaints of the low back originating from the left sacroiliac joint frequently occur in competitive inline speedskaters.

## Background

Much has been written about injuries associated with falls or collisions in recreational inline-skating [1-5], but so far no attention has been put on non-traumatic musculoskeletal complaints. In fact, very little research has been conducted at all concerning sport specific complaints of whatever cause in inline-speedskaters.

Inline speedskating is one of the fastest growing disciplines in competitive skating worldwide and races are held in a variety of formats and on a variety of surfaces. The competitive inline speedskating combines a movement pattern similar to speedskating on ice and involves pack-oriented competition modes known from cycling [6]. The competitions are generally held at roller skating rinks with oval tracks of about 100 m in circumference. Races involve the athletes attempting to establish the best time while racing counter-clockwise around the track [7].

In order to prevent future non-traumatic injuries, epidemiologic studies are needed to determine the magnitude of such injuries found within elite in-line speedskaters. Previous general observations by coach and medical staff observed frequent low back complaints which appeared to show SI involvement. The coach of the national speedskating team has expressed an interest in documenting the impact of these injuries in order to investigate this assumption and develop recommendations for rehabilitation and prevention.

We aimed at investigating whether the SI joint is a pain source of speedskaters presenting with low pain. To our knowledge, this is the first study documenting non-traumatic musculoskeletal complaints involving the lumbo-sacral area in inline-speedskaters.

## Materials and methods

The involved practitioners were invited by the German inline-speedskating national team to provide chiropractic services for both staff and athletes. Three sport events were included for data collection: 1) European Championships 2010, 2) Annual Cadre Training 2010 and 3) European Championships 2011.

The data collection for this study was conducted prior to any potential therapeutic interventions. Due to the unpredictable onset of the complaints, no protocol could be established for when the examinations were to be conducted (e.g. pre- or post competition). Also, usually no full physical examination was performed as time restraints would often only allow for a focused regional examination.

The locations for the examinations varied both between and during the events. Tests were conducted on-site in the open or in locker rooms. There were no specific procedures as to when and how the athletes were to present to the chiropractor. They did so either at the advice of a member of staff or self-referred at a time of their choosing.

Before the examination of the athletes, consent to participate in this study was obtained. As this was an observational study only and examinations were conducted irrespective of the conduction of this study, no ethics approval was sought. In addition, German regulations do not require ethics approval for this type of research [8]. However, all research was conducted in accordance with the Declaration of Helsinki on Ethical Principles for Medical Research Involving Human Subjects.

### Participants

Physical examination findings of all German athletes presenting to the chiropractor with pain in the lumbo-sacral area of non-traumatic origin were included in this study. For the purpose of this study, non-traumatic origin was defined as pain or discomfort not directly associated with a previous traumatic event such as collisions or falls. Athletes were excluded if pain radiated below the gluteal folds or there were other complaints suggesting neurological impairments (e.g. paraesthesia or muscle weakness).

As this study aimed to investigate possible SI involvement irrespective of any previous history of similar or identical complaints, findings of participants that had been examined at an earlier sport event were also included. However, regardless of the number of complaints only the first low back examination per event was to be included.

### Procedures

The pain intensity was assessed by means of a 100 mm VAS scale that the athlete filled out prior to the physical examinations.

Based on a literature search we used a protocol to test different aspects of the SI joint consisting of Gaenslen's [9], distraction, thigh thrust, compression and sacral thrust tests as their validity has been previously demonstrated [9-11].

At each of the three occasions the orthopedic testing was conducted by an experienced chiropractor (TB and AH) of the German Chiropractic Sports Council. Prior to assessing the symptomatic athletes, all procedures were practiced on 5 individuals to ensure that the involved practitioners were performing the tests in an identical fashion. The inter- or intra-rater reliability was not assessed.

The tests were conducted in the same order each time and bilaterally, starting with the symptomatic side. In order to minimize overstressing of the involved structures and thereby provoking false positives due to repetitive testing, each maneuver was immediately stopped if pain was produced. The chiropractor then proceeded with the next test (Table 1).

**Table 1:** Procedures for testing the sacroiliac joint

Test		Description
**1**	**Distraction**	The participant is positioned supine while pressure is applied to the anterior superior iliac spine directed posteriorly and laterally [23].

**2**	**Thigh thrust**	The participant is lying supine, the hip is flexed to 90° and the knee flexed. A posterior shearing stress is applied along the line of the femur [23].

**3**	**Compression**	The participant is side-lying facing away from the examiner. Downward pressure is applied to the upper most iliac crest [23].

**4**	**Sacral thrust**	The participant is lying prone while three thrusts were applied to the sacrum in an anterior direction [23].

**5**	**Gaenslen**	The participant is asked to lie supine on the edge of a table. The leg being tested is hyperextended at the hip so that it hung over the table. The other leg is flexed at the hip and knee. The participant is instructed to hold the non-tested leg with both arms while the the pelvis is stabilized by the examiner and passive pressure is applied to the tested leg to hold it in the hyperextended position. The examiner then applies additional pressure to place the hip into a position of further extension and adduction. A positive test was noted if pain was provoked or reproduced [24].

Involvement of the SI joint was assumed if a minimum of three out of five tests were positive on the same side.

The examination findings were documented as either "positive" or "negative" with regards to reproducing familiar pain. A response was noted as "unclear" if the athlete could decide whether a test reproduced pain or not. For the statistical analysis, such a result was counted as a negative response.

### Statistical analysis

Simple descriptive statistics were applied for patient demographics and the reported pain intensities. Results were expressed as means, standard deviations (SD) and, where appropriate, 95% confidence intervals (CI).

All statistical analysis was performed with IBM SPSS 19 for Windows (SPSS Inc, Chicago, USA).

## Results

### Participants

Over the course of 12 months and three events a total of 37 examinations were conducted on 34 athletes. Of these, three speedskaters presented with low back pain at both European Championships.

A total of 27 German athletes participated in the 2010 European Championships. Twelve of these (44%) visited the chiropractor because of low back discomfort. Of the 25 participating speedskaters in the 2010 cadre training camp, eleven (40%) reported lumbo-sacral complaints and participated in this study. During the 2011 European Championships 14 of the 16 participating athletes reported low back pain (88%).

Each participant had been skating competitively for around 5 years. The characteristics of the participants and the reported pain intensities are shown in Table 2.

**Table 2:** Participant characteristics and pain intensity

		Sport event	
	**European Championships 2010**	**Cadre training camp 2010**	**European Championships 2011**

**Number of patients**	12	11	14

**Age **(years)	19.8 ± 4.2 (17.5-22.1)	16.1 ± 1.7 (15.2-17.0)	20.4 ± 3.6 (18.5-22.3)

**Gender**			

Female	6	7	7

Male	6	4	7

**Pain intensity **(VAS)	35.1 ± 19.2 24.5-45.7	29.4 ± 16.8 (20.6-38.3)	23.4 ± 13.4 (16.2-30.50)

VAS: visual analogue scale

All values are mean ± SD (95% CI)

Four athletes were assessed at two separate events by different examiners. The reported pain intensities ranged from mild to moderate pain [12] (VAS 23.4 ± 13.4 to 35.1 ± 19.2) pre-examination and kept none of the athletes from competing in the events.

Participants primarily described a tension in the lumbo-sacral region or unilateral pain in this area of non-traumatic origin that was not radiating beyond the gluteal folds.

### Physical examination findings

All athletes were able to tolerate the examination procedures and there was no trend towards an increased likelihood of positive findings towards the end of the testing sequence.

Apart from a single athlete, all other participants 2010 European Championships with pain in the lumbo-sacral region showed SI involvement (11/12, 92%). In the vast majority of these cases, the left side was involved (9/11, 82%). Of the 11 speedskaters at the Cadre training camp 2010 with a symptomatic low back, two athletes did not show evidence of SI involvement. Where the tests suggested the SI joint as the source of pain (9/11, 82%), in all but two the left side was involved (8/9, 89%). The team for the 2011 European Championships consisted of 16 athletes. Of the 14 patients with pain in the SI area, all but three presented with SI involvement (11/14, 79%). Where the physical examination pointed towards SI involvement, the left side was identified in 91% (10/11) of cases (Table 3).

**Table 3:** Physical examination findings

**Athlete No**.	Side involved		Provocative tests				Number of positive tests
			
		Distraction	Thigh Thrust		Sacral thrust	Gaenslen	
**European Championships 2010**							

01	Left	-	-	+	+	+	3

02	Left	+	+	-	+	-	3

03	Right	+	+		+	+	4

04	Left	+	+	+	+	+	5

05	Left	-	-	-	+	unclear	(1)

06	Left	+	+	-	+	-	3

07	Left	+	+	+	+	+	5

08	Right		+	+		+	3

09	Left	+	+		+	+	4

10	Left	+	-	+	-	+	3

11	Left	+	+	+		+	4

12	Left	-	+	+	+	-	3

		**Cadre training camp 2010**					

13	Left	+	+		+	-	3

14	Left	-	-	+	-	-	(1)

15	Left	+	+	+		+	4

16	Right	+	+	-	+	-	3

17	Left		+	+	+	+	4

18	Left	+	+	+	-	-	3

19	Left	-	+	-	+	+	3

20	Left	-	-	+	-	-	(1)

21	Left	+	+	unclear	+	+	4

22	Left		+	+		+	3

23	Left	+	+	-	+	-	3

		**European Championships 2011**					

24	Left	+	+	-	+	-	3

25	Left	+	-	-	+	+	3

26	Left	-	-	+	-	-	(1)

27	Right	+	+	+	+	+	5

28	Left	+	+	+	-	-	3

29	Left	-	+	+	+	-	3

30	Left	-	+	-	+	+	3

31	Left	-	-	-	-	-	(0)

32	Left	-	-	+	+	+	3

33	Left	+	+	+	+	+	5

34	Right	-	-	-	-	+	(1)

35	Left	+	+	+	+	+	5

36	Left	+	+	-	+	-	3

37	Left	+	+	-	+	-	3

-:negative,+ : positive, n = number

Overall, of those athletes presenting with unilateral pain in the lumbo-sacral area during these three events, about 90% (31/34, 91%) originated from the SI origin, with 87% (27/31) presenting with left sided involvement. None of the speedskaters described bilateral symptoms or had bilaterally positive examination findings.

## Discussion

This study indicates that the SI joints were frequently involved in this cohort of elite speedskaters with non-traumatic low back pain whereby the left side was affected in the vast majority of cases. The study design did also not allow prevalence estimates.

So far there is no universally accepted gold standard for the diagnosis of pain originating from the lumbar spine or sacroiliac (SI) joint but anesthetic or provocative injections have been recommended by some pain physicians [13]. However, these procedures are invasive and expensive, and therefore may not be suitable for routine on-site clinical use. In addition, their validity has been questioned as well [14]. As local anesthetic blocks could not be employed for this study, we relied on orthopedic provocative maneuvers instead.

At first sight it appears reasonable to assume that simply stressing a joint should provoke pain of this origin. However, such provocative tests are problematic as it is unlikely that only the intended structure is loaded in isolation. Consequently, if a familiar pain is reproduced by such a stress test, it remains unclear if this is evidence that the targeted structure alone is involved or whether another nearby one that has been stressed simultaneously [14]. If, however, several positive findings of a test cluster were to be combined, greater diagnostic confidence is permitted.

While these points have to be kept in mind, diagnostic procedures feasible for on-site clinical application have to be selected in order to establish a diagnosis as a basis for any type of intervention.

After several studies with inconclusive or conflicting results [11], a recent study investigating the reliability of motion- and pain provocation tests for the sacroiliac (SI) joint concluded that a combination of three out of five tests should be employed for acceptable reliability [15]. With regards to validity, Lasett et al. found that performing the distraction, thigh thrust, compression and sacral thrust tests offered the best diagnostic value for SI restrictions. Two positive tests were the best predictors of a positive intra-articular SI block [10]. We used a combination of these recommendations.

Overall, a high percentage of athletes complained about low to medium intensity SI symptoms with the left side being predominately involved. For those studies with negative SI tests, other structures such as muscles, discs, facet joints or other structures of the kinematic chain may be suspected as the pain source. As we did not specifically test for these structures, we are unable to comment on this.

While this points towards an increased biomechanical stress and demand on these structures, the high number of cases presenting with a low pain intensity may be attributed to the convenience of having chiropractic services available on site.

The fact that the majority of athletes participating in the 2011 European Championships presented with SI symptoms does therefore not necessarily point towards a higher number of cases. Instead, the observed trend may simply be attributed to a higher utilization of chiropractic services as athletes got familiar with it.

As the low average pain intensities particularly at this event suggest, athletes may have routinely presented to the chiropractor with only minor discomfort or tension for which they normally may not have sought medical attention. This can be attributed to the fact that even minor feelings of discomfort may adversely affect performance during competition. Consequently, it is difficult to determine from our data the athletes affected by truly clinically relevant SI complaints.

The high percentage of positive SI tests may be surprising considering the reported low pain intensity scores. The examiners took care to challenge the SI joint just sufficiently to reproduce the symptoms. However, while less force/stress may have been required at higher pain intensities, nociceptive input from surrounding structures such as muscles may have also increased the possibility of false positives. In fact, it appears unlikely that the SI joint is solely responsible for symptoms without surrounding structures being involved to some degree [16]. In addition, other factors that we may not have tested for in all athletes, such as disc or spinal pathologies, could have contributed to positive SI-test findings.

In addition, the possibility of false positives cannot be excluded as the sequence of five tests may have stressed the area sufficiently to produce pain. However, as the testing sequence remained identical and no trend towards more positive tests were observed with later tests, it may be assumed that the results remained unaffected by repetitive testing.

While the study design does not allow any concluding on causation, the results nevertheless tempt us to hypothesize that the force vectors associated with counter-clockwise track orientation may play a major role. While ground reaction forces in speed skating remain relatively low, maximal moments at the hip are high and reach values of 140-160 Nm when skating in the straight part of the track [17]. As the athlete is leaning inwards to follow the curves in counter-clockwise direction, even greater values are expected on hips and, in extension, the SI joints. It has been further shown that in ice speedskaters higher loads are applied to the left leg when skating the curves compared to the right leg [18]. This may therefore explain the predominantly left sided symptoms.

Although similar biomechanics apply, it remains unclear whether the findings presented here can also be transferred to speedskating on ice and further studies are needed to investigate this.

### Implications for training and rehabilitation

The testing procedures did not allow any conclusion on the causative nature of the SI symptoms. However, routine self-mobilization procedures [19] for the SI joint may be indicated unless the pain is associated with an inflammatory process due to overuse rather than joint hypomobility.

As previously discussed, it cannot be excluded that the other structures such as the sacroiliac or sacrotuberal ligaments contributed to the reported symptoms and positive test findings. However, structures directly surrounding the SI joint will most likely be addressed as well during a therapeutic intervention or rehabilitation as any true isolation of a desired structure appears impossible.

In addition, balancing agonistic and antagonistic lower extremity muscles with regards to length and strength is an important preventive measure as direct and indirect force transmission occurs across the sacrum and ilium [20]. Muscles found to be affected include the iliopsoas, rectus femoris, tensor fascia lata, latissimus dorsi, hip adductors [21], gluteus and quadratus lumborum [16,21], as well as the piriformis muscle [20].

Although maintaining individual muscle flexibility and strength is important, retraining functional coordination between multiple muscle groups is a key to a recovery. This can for example be facilitated by individually targeted lumbo-pelvic stabilization exercises, proprioceptive training as well as sport-specific activities [22].

### Strengths and limitations

The cluster consisting of five tests for the SI joints constitutes a major strength of this study with regards to the validity of the results.

Unfortunately, the nature of such field observations as described here entails certain limitations. Although the causality cannot be predicted, the SI joint findings may appear to be a result from overuse associated with training or competitions. It is therefore not feasible to assess such participants in a controlled, non-competitive clinical setting. Further complications are associated with the low number of elite athletes available and the fact that the members of the national team are located all across Germany.

Secondly, due to time restraints between competitions and training sessions, no extended health questionnaires could be distributed nor full physical examinations conducted on all occasions. This may have resulted in bias as previous injuries or treatments may have affected the results of this study. However, the athletes were asymptomatic prior to the competitions as they otherwise would not have been on the team. This indicates that the presenting complaints most likely occurred in association with practice or races at the respective event.

Thirdly, some athletes may have visited the chiropractors immediately after symptoms were noticed, whereas others would have arrived later on. Also, some of the speedskaters were examined right after an exhausting competition that may have initiated the symptoms (with all implications regarding fatigue, muscle tone etc.) whereas others would have rested between training sessions or competitions. This means that physical examinations were not performed under the same overall conditions which in turn may have affected the results for both pain intensity and examination findings. However, this is what the sports physician has to accommodate with, including limited time available for the examinations and treatments that have to be scheduled around competitions and training. Although desirable, these time restraints also prohibited a broader, more detailed musculoskeletal examination cluster to be conducted with each athlete that would have allowed commenting on other biomechanical deficits.

In addition, a high risk of selection bias was unavoidable, e.g. with regards to the small group of athletes, which rendered prevalence estimates problematic. Despite all intentions to test objectively, it is also possible that the involved chiropractors may have been biased towards detecting positive SI-joint tests as previous experience suggested such findings. However, it may be concluded nevertheless that the left SI joint was commonly involved in those presenting with low back pain.

In order to reduce the risk of bias, future studies may want to conduct more detailed examinations including spine and lower extremities. This would allow commenting on other potential pain generating structures that may further contribute to positive SI-joint findings. Examiners otherwise not involved in this sport and blinded to our results may also be beneficial. However, as mentioned before, such time consuming procedures may not be feasible during competitive events. In addition, it may be difficult to gather such elite athletes in a more controlled setting and test them during practice sessions where a reduced willingness to perform may not elicit similar results.

## Conclusion

Non-traumatic pain or discomfort in the lumbo-sacral region was commonly encountered in this group of elite inline speedskaters. In the vast majority of the participants, the SI joint appears to be the origin of the complaint, of which the left side is involved in nearly 90% of cases. We hypothesize that this may be associated with increased loading on this leg when skating the curves.

## Competing interests

The authors declare that they have no competing interests.

## Authors' contributions

AR conducted the statistical analysis and drafted the manuscript. TB and AH conducted the physical examinations and assisted in drafting the manuscript. All authors read and approved the final manuscript.
